# Editorial: Neuroimaging and Neuropsychology of Meditation States

**DOI:** 10.3389/fpsyg.2015.01757

**Published:** 2015-11-19

**Authors:** Barbara Tomasino, Franco Fabbro

**Affiliations:** ^1^Department of Human Sciences, University of UdineUdine, Italy; ^2^Department of Medical and Biological Sciences, University of UdineUdine, Italy; ^3^Perceptual Robotics Laboratory, Sant'Anna School of Advanced StudiesPisa, Italy

**Keywords:** meditation, expertise, neuropsychology, fMRI, meta-analysis, attention

One way of training cognitive functions and triggering plasticity can be through exercising meditation (Barinaga, 2003; Knight, 2004). Accordingly, it has been shown that this complex cognitive state induces both neurophysiological and psychological modifications, which have been consistently addressed by neuroscience regarding their potential benefit for mental and physical health (Davidson and McEwen, 2012).

In particular, the brain network governing meditation has been studied using a variety of strategies eliciting different cognitive processes (e.g., silence, attention to own body, sense of joy, mantras, etc.). Furthermore, the effect of expertise (i.e., short- vs. long-term) has been shown to influence the areas activated by meditation. Lastly, meditation training has been found to influence cognitive performance, e.g., attention, executive functions.

In order to promote the development of the neuroscientific investigation on how meditation activates and can modify brain areas, this Frontiers Research Topic aimed at bringing together studies from groups of authors whose research focus on neural mechanisms involved in meditation.

We collected fifteen contributions addressing this issue from a neuroimaging and a neuropsychological perspective. We solicited studies addressing meditation-related changes by using functional imaging (*fMRI*), resting state (*default mode network*), structural analyses (e.g., voxel based morphometry, *VBM*) as well as functional connectivity and structural connectivity (diffusion tensor imaging, *DTI*). In addition, we collected studies in which behavioral and neuropsychological studies in which meditation techniques have been used and in which cognitive changes have been found. Lastly, we included studies in social-affective neuroscience, reporting meditation-related modifications of personality, or changes of emotion-related network regulating stress and cognitive resource.

There are three activation likehood estimation (ALE) meta-analyses on studies using PET, SPECT, or fMRI techniques in which subjects performed meditation. In the first study (Sperduti et al., 2011) analyzed 10 studies and reported significant activations in the basal ganglia, the enthorinal cortex, and the medial prefrontal cortex. In a second study (Tomasino et al., 2012) analyzed 24 experiments and 150 activation foci which were divided according to different aspects of meditation (focused attention, mantra and the effects of experience). The meta-analysis evidence that meditation based on focus attention activates a network involving the medial gyrus bilaterally, the left superior parietal lobe, the left insula, and the right supramarginal gyrus (SMG). Mantra based meditation activates the right SMG, the SMA bilaterally and the left postcentral gyrus. The effect of meditation experience influenced the meditation network of activation. Expert meditators as compared to those with a short meditation experience had an increase activation in posterior areas such as the right SMG, whereas short-term experienced meditators has an increased frontal lobe activation.

In an another study (Tomasino et al., 2014) performer an ALE meta-analysis on meditation studies divided according to whether they were inspired to Buddhist (16 experiments, 263 subjects, and 96 activation foci) or to Hinduism traditions (8 experiments, 54 activation foci, and 66 subjects). The first type of meditation enhanced activations in some frontal lobe structures associated with executive attention, possibly confirming the fundamental role of mindfulness shared by many Buddhist meditations [see for a recent review on mindfulness meditation (Tang et al., 2015a)]. By contrast, the network related to Hinduism-inspired meditation triggered a left lateralized network of areas including the postcentral gyrus, the superior parietal lobe, the hippocampus and the right middle cingulate cortex. The dissociation between anterior and posterior networks support the notion that different meditation styles and traditions are characterized by different patterns of neural activation. These meta-analyses evidenced the importance of specifying the type of meditation under study and the spiritual tradition from which it derives, thus particular care has been taken in the present special topic in defining in each contribution the type of meditation which has been studied.

The forms of meditation inspired to Buddhist tradition, and in particular those involving mindfulness, are related to voluntary attention (*anapanasati*), to body awareness (body scan) and to the observation of the mind (*vipassana*) and are correlated to activation of the executive frontal system, of some part of the insula and of medial frontal structures (Tomasino et al., 2012, 2014). The forms of meditation inspired to Hinduism tradition, as well as subjects performing meditation inspired to Buddhism tradition with a long-term meditation experience, have the aim of reaching *samadhi*, a condition characterized by an emptying state of consciousness [this concept has been discussed in Berensten's opinion article (Baerentsen, 2015)], who has recently been classified as an enhanced non-cognitive/non-affective state (NC/NA) (Nash and Newberg, 2013). These forms of meditation are related to activation/deactivation of posterior areas, in particular of the right supramarginal gyrus (Tomasino et al., 2014), a brain area related to out-of-body-experience phenomena (Blanke et al., 2002, 2004; Blanke and Mohr, 2005) and auto-transcendence (Urgesi et al., 2010; Crescentini et al., 2014).

From a practical point of view is useful classifying the different forms of meditation [see for instance Lippelet et al.'s mini review article (Lippelt et al., 2014)], this working strategy is not so straight. Hasenkamp et al. (2012) and Malinowski (2013) evidenced how mindfulness meditation is a process in which different phases are continuously reached (e.g., focus on breathing, mind wanders, recognize wandering, letting go, return to the task) and each of these phases is correlated with activation or deactivation of different brain networks. This logic is valid also for the other forms of meditation either those inspired to Buddhist and those inspired to Hinduism traditions. In light of this observation, the network analysis techniques proposed in the Special topic by Kemmer et al. (2015) in an original research article looks very promising.

Numerous experimental studies and revisions presented in this issue reaffirmed the beneficial effects on the psychological and clinical level of meditation trainings or meditation practice see Simon and Engström (2015) in a perspective article on the positive effects of meditation in treating psychological disorders; Sun et al. (2015) in a review article, on the effects of meditation on decision making; Mascaro et al. (2015) in an hypothesis and theory article on how meditation can enhance prosocial emotions; McConnell et al. (2014) in an original research article, on the use of binaural beats; Tang et al. (2015b) in an original research article on the effect of 5-h integrative body-mind training on frontal asymmetry; Fan et al. (2015) in an original research article on the effect of 5-h integrative body-mind training on conflict resolution; and Kirk and Montague (2015) in an original research article on self-control in Buddhist meditators. Crescentini and Capurso (2015) in a mini review article summarized the results of studies in which the effects of mindfulness meditation training had an impact on personality profiles and self-concepts. Authors argue that meditation can shape in addition to attention regulation, emotional regulation and body awareness, also subjects' personality toward more healthy functioning by transforming habitual patterns of responding.

In particular, Nakata et al. (2014) in a review article addressed the mechanisms involved in the use of meditation in pain control. They reported that several studies on meditation showed an increase of activation in brain structures related to psychophysiological pain processing, instead of a reduction. The authors referred to this inconsistency of results by using the following expression “the mystery of meditation.” In our view, the increase of activation could be related to a general attitude in meditation inspired to Buddhist tradition. According to Buddha's indications the most correct behavior does not correspond to pain reduction or avoidance, but in facing the experience of pain through meditation. Indeed escaping and avoidance of pain-related experience increase suffering, whereas facing with mindfulness the experience of pain although activates the brain circuits related to pain processing, causes a decrease of suffering (Fabbro and Crescentini, 2014).

Other studies discussed the structural brain changes induced by meditation trainings. It has been shown that brief meditation trainings could increment white matter pathways connecting the anterior cingulate cortex to other brain areas. Posner et al. (2014) in an hypothesis and theory article put forward an hypothesis about the molecular mechanisms that could be the basis of white matter changed. Luders et al. (2015) in an original research article evidenced how meditation could trigger an increase of gray matter in the hippocampus with differences in male and female meditators. Furthermore, the same group (Luders et al., 2014) in a second original research article performed a study on a large sample of adult meditators and showed that the decrease of gray matter that occurs with aging could be significantly slowed in meditators as compared to non-meditators. This result indicates that meditation, as well as bilingualism, is a factor that favoring the cognitive reserve, both in neurological diseases and in aging.

Unfortunately the present special topic lacks of neuropsychological studies in which meditation has been used as a training with neurological patients. We believe that this leaves open the debate on meditation potential brain effects and could be subject of a future special issue.

## Conflict of interest statement

The authors declare that the research was conducted in the absence of any commercial or financial relationships that could be construed as a potential conflict of interest.
